# Intelligent Evaluation of Global Spinal Alignment by a Decentralized Convolutional Neural Network

**DOI:** 10.1007/s10278-021-00533-3

**Published:** 2022-01-21

**Authors:** Thong Phi Nguyen, Ji Won Jung, Yong Jin Yoo, Sung Hoon Choi, Jonghun Yoon

**Affiliations:** 1 Department of Mechanical Engineering, BK21 FOUR ERICA-ACE Centre, Hanyang University, 55 Hanyangdaehak-ro, Sangnok-gu, Ansan, Gyeonggi 15588 Republic of Korea; 2 Department of Orthopaedic Surgery, Hanyang University College of Medicine, 222 Wangsimni-ro, Seongdong-gu, Seoul, 04763 Republic of Korea; 3 Department of Mechanical Engineering, Hanyang University, 55, Hanyangdaehak-ro, Sangnok-gu, Gyeonggi-do, Ansan-si, 15588 Republic of Korea

**Keywords:** Radiology, Spinopelvic, Artificial intelligent, Orthopedic, Convolutional neural network

## Abstract

Degenerative changes of the spine can cause spinal misalignment, with part of the spine arching beyond normal limits or moving in an incorrect direction, potentially resulting in back pain and significantly limiting a person’s mobility. The most important parameters related to spinal misalignment include pelvic incidence, pelvic tilt, lumbar lordosis, thoracic kyphosis, and cervical lordosis. As a general rule, alignment of the spine for diagnosis and surgical treatment is estimated based on geometrical parameters measured manually by experienced doctors. However, these measurements consume the time and effort of experts to perform repetitive tasks that could be automated, especially with the powerful support of current artificial intelligence techniques. This paper focuses on creation of a decentralized convolutional neural network to precisely measure 12 spinal alignment parameters. Specifically, this method is based on detecting regions of interest with its dimensions that decrease by three orders of magnitude to focus on the necessary region to provide the output as key points. Using these key points, parameters representing spinal alignment are calculated. The quality of the method’s performance, which is the consistency of the measurement results with manual measurement, is validated by 30 test cases and shows 10 of 12 parameters with a correlation coefficient > 0.8, with pelvic tilt having the smallest absolute deviation of 1.156°.

## Introduction

Lumbar lordosis is the normal inward curvature of the lumbar spine observed only in human beings. It supports the body, absorbs shock, and ensures stability and flexibility, maintaining the torso within the cone of economy of so-called genuine bipedalism [1]. When the curve arches too far inward, it is known as increased lumbar lordosis or swayback disease [2]. Abnormal kyphosis is a spinal disorder caused by excessive outward curve of the thoracic spine and is referred to as Scheuermann’s kyphosis [3]. In the cervical region of the spine, there is also dropped head syndrome, in which the neck bends forward too much to maintain a horizontal gaze [4]. It is well known that sagittal spinal deformities are more associated with low quality of life and debilitating conditions than is deformities in the coronal plane [5]. There have been many studies to determine the range of angles that represent the alignment status of spinal regions via geometrical relationships [6–8].

In data collection procedures, geometrical parameters often are manually measured from X-ray images by experienced surgeons. This can help limiting data collection errors and consequently increasing the reliability of analyses drawn from the dataset. However, it consequently requires a lot of effort and time, especially in research studies that require diverse datasets. To measure an angle parameter, such as a Cobb angle between two vertebrae, the radiologist need to draw tangent lines along the end of vertebrae on X-ray images by a ruler and a pencil, and then measure the angle using a protractor. This procedure highly dependents on the radiologist’s experience and also consumes a lot of time because of the large number of angle parameter between vertebras in one spine. Also, manual measurement of parameters presents a significant challenge in aggregating data for statistical analysis which is mostly performed sequentially by hand.

Due to the development of artificial intelligence techniques, especially regarding deep learning, such applications in medical metrology that use vision-based data types have significant potential. One of the strengths of this technique is that it reduces the labor of repetitive work through a combination of the high-volume processing power of computers and the ability of artificial intelligence techniques to generate a set of weighting factors optimized to simulate the experience of experts. In addition, data collection after automatic measurement can be conveniently integrated with statistical tools, especially for large-scale datasets. Aubert et al. [9] proposed a method to automatically reconstruct the spine in three dimensions, in which a realistic statistical model of the spine was fitted to images using a convolutional neural network (CNN). Weng et al. [10] introduced an approach that used regression deep learning to estimate automatically the sagittal vertical axis, which is one of the parameters that describes sagittal alignment. Cho et al. [11] presented a study using U-net to segment the landmarks in radiographs and subsequently measure key parameters including cervical lordosis (CL), thoracic kyphosis (TK), pelvic incidence minus lumbar lordosis (PI-LL), sagittal vertical axis (SVA), and pelvic tilt (PT). Wu et al. [12] proposed a novel Multi-View Correlation Network architecture to measure the Cobb angle according to detected landmarks but did not mention parameters for sagittal alignment. For automatic measurement of parameters representing the state of spinal alignment in this study, a program was developed based on a decentralized CNN, which was introduced by Chae et al. [13] and Nguyen et al. [14]. The program was highly rated in terms of correlation with manual measurements. However, both of these mentioned methods were mostly developed for positioning only the lumbar and sacrum regions of the spine, while the symptoms of swayback disease or abnormal kyphosis requires, also, the detection of vertebras on cervical regions. Therefore, it requires an upgraded method that the model provides the ability to evaluate global spinal alignment.

Most research focuses only on localized spinal regions, especially the lumbar region, while there is no comprehensive research on automatic measurement methods to evaluate global spinal alignment. Increased measurement of geometrical parameters of the entire spine could provide information for simultaneous monitoring and diagnosis of spinal sagittal deformities. This paper proposes a method that uses decentralized CNN algorithms to locate required key points and consequently measure the spinal alignment based on 12 parameters distributed throughout the cervical, thoracic, and lumbar spine. The measurement accuracy of the proposed method is validated via a comparison with manual measurements from experienced doctors as standard references.

## Methods and Materials

### Representative Parameters for Spinal Alignment

**Fig. 1:**
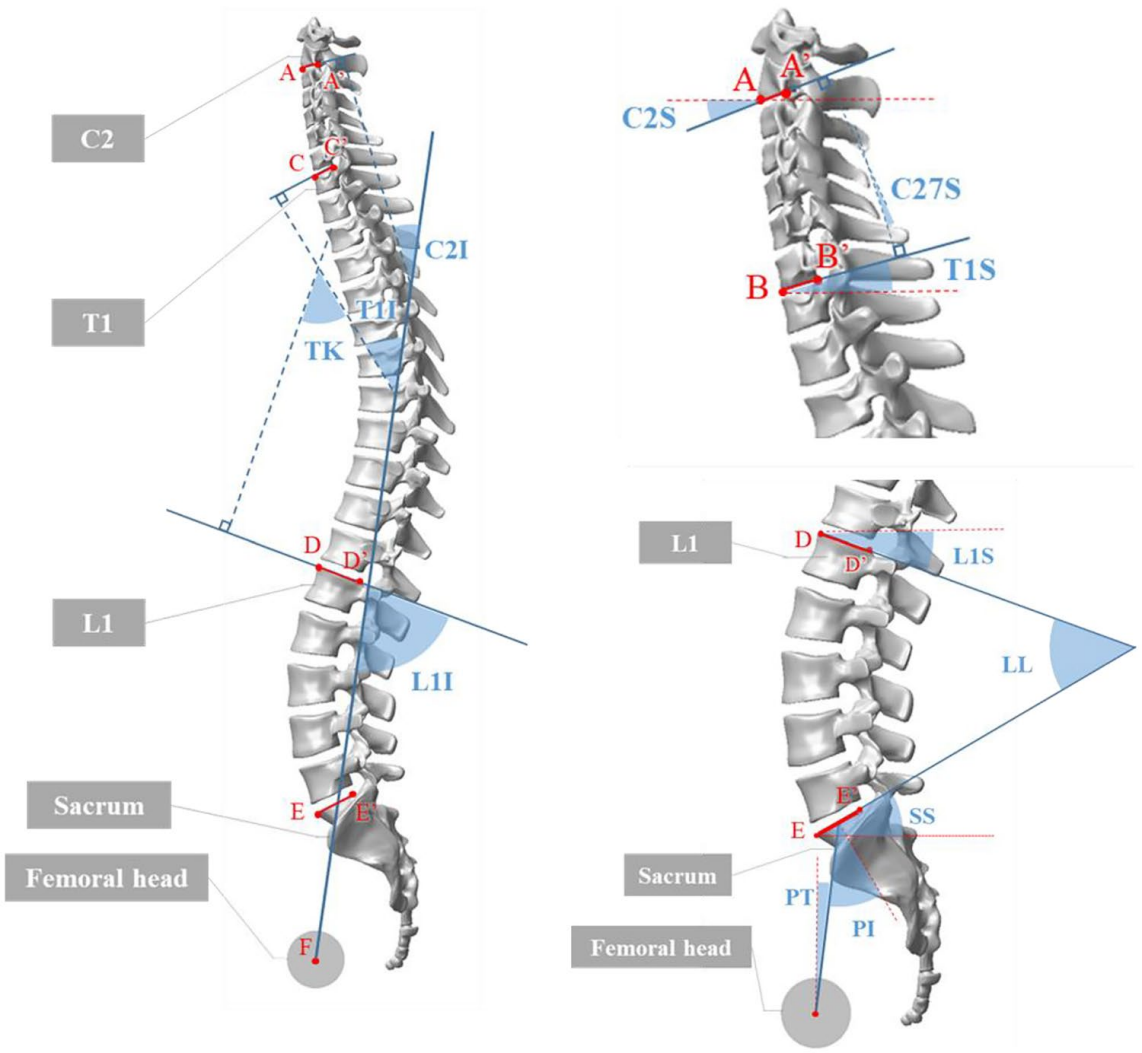
Representative parameters of spinal sagittal alignment

The angles chosen to characterize spinal alignment are determined according to geometrical relationships between quantities of interests, as shown in Fig. 1, including C2 lower endplate (line AA’), C7 lower endplate (line BB’), T1 superior endplate (line CC’), L1 superior endplate (line DD’), sacrum superior endplate (line EE’), and the center of the femoral head (point F). Based on these points of interest positioned in spinal sagittal X-rays, representative parameters are measured.

1. Pelvic incidence (PI) was defined as the angle between a line drawn from the center of the femoral heads (point F) to the midpoint of the sacral superior endplate (line EE’) perpendicular to the sacral superior endplate.
2. Pelvic tilt (PT) was defined as the angle between a line drawn from the center of the femoral heads (point F) to the midpoint of the sacral superior endplate (line EE’) and the vertical line.
3. Sacral slope (SS) was defined as the angle between the sacral superior endplate (line EE’) and the horizontal line.
4. Lumbar lordosis (LL) was defined as the angle between the superior endplate of L1 (line DD’) and the superior endplate of S1 (line EE’).
5. L1 incidence (L1I) was defined as the angle between a line drawn from the center of the femoral heads (point F) to the midpoint of the sacral superior endplate (line EE’) and perpendicular to the L1 superior endplate [15].
6. T1 incidence (T1I) was defined as the angle between a line drawn from the center of the femoral heads (point F) to the midpoint of the sacral superior endplate (line EE’) and perpendicular to the T1 superior endplate.
7. C2 incidence (C2I) was defined as the angle between a line drawn from the center of the femoral heads (point F) to the midpoint of the sacral superior endplate (line EE’) and perpendicular to the C2 inferior endplate [15].
8. Thoracic kyphosis (TK) was defined as the angle between the superior endplate of T1 (line CC’) and the superior endplate of L1 (line DD’).
9. C2-C7 lordosis (C27L) was defined as the angle between the inferior endplate of C2 (line AA’) and the inferior endplate of C7 (line BB’).
10. L1 slope (L1S) was defined as the angle between the horizontal line and superior endplates of L1 (line DD’).
11. T1 slope (T1S) was defined as the angle between the horizontal line and superior endplates of T1 (line BB’).
12. C2 slope (C2S) was defined as the angle between the horizontal line and inferior endplates of C2 (line AA’).

In an approach that differs from determining the lower and superior endplates of typical vertebrae (from C3 to L1), which is based on pairs of upper or lower corners and can be applied for special vertebrae such as C2 and S1, and the representative line AA’ is drawn from the anterior and posterior corner of C2 inferior endplate (Fig. 1).

### X-ray Image Preparation

The X-ray images for parameter measurement included 500 whole spine lateral radiographs from 500 patients obtained from March 2019 to November 2020 at a single institute. The collected radiographs were obtained with different X-ray scanners that had the average pixel dimensions of 3240 × 1080. To increase the diversity of the constructed dataset, both good-posture cases and abnormal spinal deformity cases are included which particularly consist of images of 283 good-posture cases, 101 lumbar lordosis cases, and 116 thoracic kyphosis cases. Furthermore, during image collecting process, the posture of patient intentionally distributed from flexion to extension view, with forward and backward bending options. Totally, it includes 150 images of spinal flexion, 200 images of spinal neutral, and 150 images of spinal extension. Besides, cases of minors with the skeleton that is not fully developed and cases of degenerative disk disease, which the corners of a vertebra are difficult to be observe, were excluded from the dataset. And only high-quality images in which the contrast and brightness were suitable for observation were used.

### AI-Based Measuring Program

Fukushima first introduced the CNN [16], which is well known for its feature auto-extracting ability via multi-types of layers. After the features are successfully extracted, a part of the fully connected layers (FC layers), which form a deep learning network with set weighting factors optimized during the training process, can predict the required information of the input image. Therefore, the variety of the training dataset is one of the important factors that determine the accuracy of the predicted outcome.

**Fig. 2:**
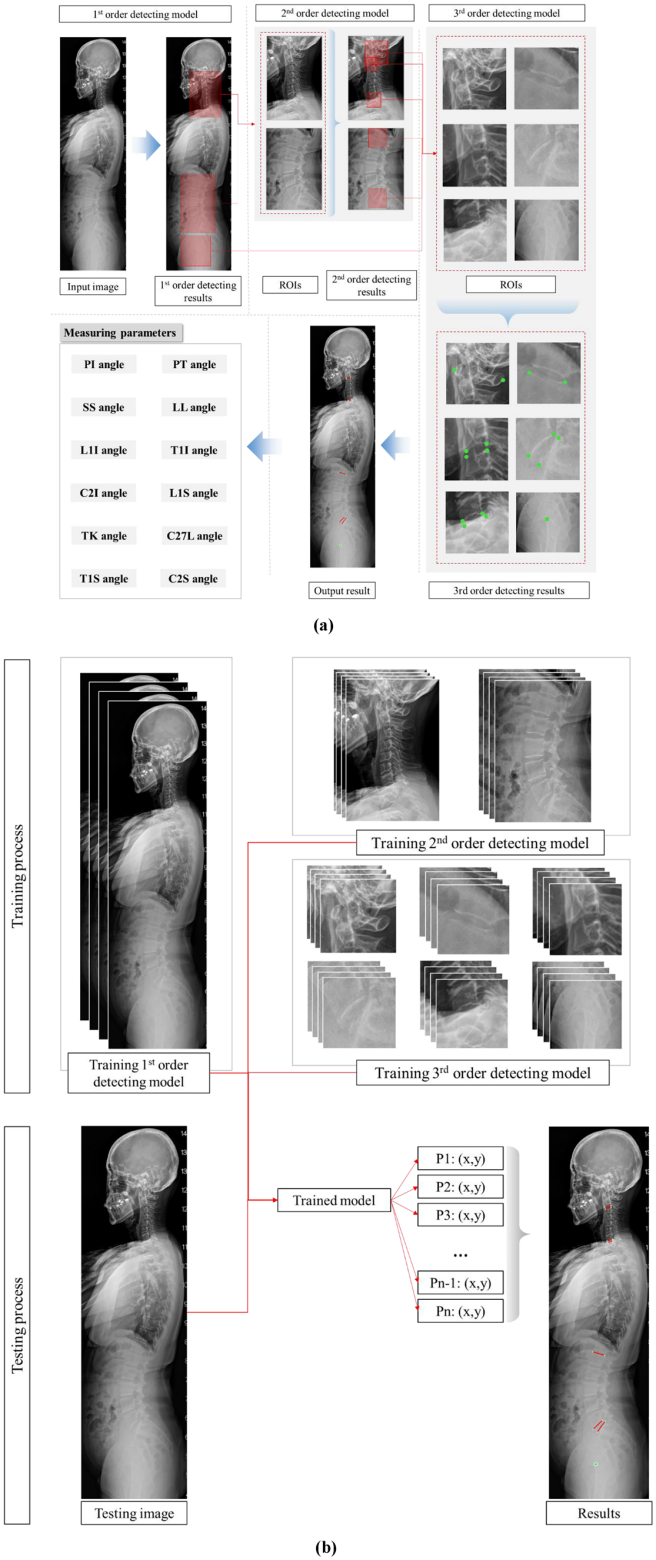
Detection procedure for the required points with the CNN model. (**a)** Partition into first, second, and third order detection. (**b)** Overall flowchart for the training and testing dataset

Consequently, a decentralized CNN provided a solution that was decentralized to multiple detecting orders to achieve high accuracy with a small number of training images, which is extremely important in medical metrology. However, different from the previous research of Chae et al. [13], which used three orders to locate the spinal region of L1–S1 vertebrae and femur head, or of Nguyen et al. [14], which located intervertebral discs between adjacent vertebrae from L1 to S1, the developed program utilized 3 detecting orders to locate key points in 3 regions of interest (ROIs) of the cervical section with C1–T1 vertebrae, the lumbar section with L1–S1 vertebrae, and the femoral heads section, which adapt to typical parameters, as shown in Fig. 2a. In detail, after the three ROIs representing the cervical spine, lumbar spine, and femoral head sections were detected by the first-order model, six minor ROIs were determined, including one for the C1 vertebra; four for the intervertebral discs in C2–C3, C7–T1, T12–L1, and L5–sacrum areas; and one for the femoral heads. At last, the exact position of each key point was calculated based on the corresponding ROIs obtained in the third order model. Figure 2b depicts the overall image training flowchart, with 500 input images having 1620 × 540 pixel resolution for training the first-order model, 8454 input images with 500 × 250 pixel resolution for training each second-order model, and 76,086 input images with 150 × 150 pixel resolution for training each third-order model. The accuracy improvement was a consequence of not only decentralized detection but also dataset increment with the application of additional augmentation techniques. Considering the sagittal X-ray images which includes cases of patients having the sight views of both left and right with the unchanged direction of the sagittal X-rays along vertical axis, flipping augmentation along horizontal axis is applied. Besides, vertebras have multiple size and orientation depending on the its position on the spine and also the individual. Therefore, the augmentation of rotation and scale are utilized to increase the diversity of the dataset for training the third-order models.

This study was approved by the institutional review board of our hospital (HYU 2020–05-032–001).

### Architectures

After the input image features are extracted by the convolution [17] and max-pooling layers [18], two fully connected (FC) layers are used to calculate the position in terms of both horizontal and vertical values. Tables 1, 2 and 3 present the dimensions of each layer along with the filter size and kernel applied for the first-, second-, and third-order detection models. However, the output number for each model depends on the quantity of required points that need to be detected in the considered ROIs. Each convolutional layer is composed of a set of matrices (filter/kernel) containing weighting factors, which are automatically updated for the features extracted during training. To reduce the output size from the previous layers, the max-pooling layers introduced select the maximum sub-matrix value as a representative element in the max-pooling matrix. After constructing the convolution and max-pooling layers, the rectified linear unit (ReLU) [19] layer targets the replacement of negative input values with a zero value, because they are unnecessary for training. From the ReLU layer results, two FC layers with optimized weighting factors pass feature extraction and are used to calculate the final results, including the position values of the required points.

**Table 1:** Specification of layer size and shape of the first-order detecting model

Layer	Filter size/kernel size	Output shape
Conv1	8 filter 9 × 9	(1612, 532, 8)
Max—pooling 1	4 × 4	(403, 133, 8)
Conv2	16 filter 7 × 7	(397, 127, 16)
Max—pooling 2	3 × 3	(132, 42, 16)
Conv3	16 filter 7 × 7	(126, 36, 16)
Max—pooling 3	2 × 2	(63, 18, 16)
Conv4	32 filter 5 × 5	(59, 14, 32)
Max—pooling 4	2 × 2	(29, 7, 32)
Conv5	32 filter 3 × 3	(27, 5, 32)
Dropout		(27, 5, 32)
ReLU		(27, 5, 32)
Fully connected 1	256	256
Fully connected 2	128	128
Output	First-order model for entire input image: 2

**Table 2:** Specification of layer size and shape of the second-order detecting model

Layer	Filter size/kernel size	Output shape
Conv1	8 filter 9 × 9	(492, 242, 8)
Max—pooling 1	3 × 3	(164, 81, 8)
Conv2	16 filter 7 × 7	(158, 75, 16)
Max—pooling 2	2 × 2	(79, 37, 16)
Conv3	16 filter 7 × 7	(73, 31, 16)
Max—pooling 3	2 × 2	(36, 15, 16)
Conv4	16 filter 5 × 5	(32, 11, 32)
Max—pooling 4	2 × 2	(16, 5, 32)
Conv5	32 filter 3 × 3	(14, 3, 32)
Dropout		
ReLU		(14, 3, 32)
Fully connected 1	256	256
Fully connected 2	90	90
Output	Second-order model for cervical region: 6 Second-order model for lumbar region: 4

**Table 3:** Specification of layer size and shape of the third-order detecting model

Layer	Filter size/kernel size	Output shape
Conv1	8 filter 9 × 9	(142, 142, 8)
Max—pooling 1	2 × 2	(71, 71, 8)
Conv2	8 filter 7 × 7	(65, 65, 8)
Max—pooling 2	2 × 2	(32, 32, 8)
Conv3	16 filter 5 × 5	(28, 28, 16)
Max—pooling 3	2 × 2	(14, 14, 16)
Conv4	16 filter 3 × 3	(12, 12, 16)
Max—pooling 4	2 × 2	(6, 6, 16)
Conv5	32 filter 1 × 1	(6, 6, 32)
Dropout		
ReLU		(6, 6, 32)
Fully connected 1	256	256
Fully connected 2	90	90
Output	Third-order model for C1, L1 region: 4 Third-order model for C2, T1, S1 region: 8 Third-order model for femoral heads region: 2	

However, a highly complex CNN structure can lead to redundancy of untrained weighting factors, which is known as overfitting [20], and tends to induce accuracy variation between the training and testing results. The dropout layer (Google et al. [21]) is proposed to eliminate random connections between the nodes of two layers. Designing a suitable CNN architecture is an essential task that not only helps to avoid underfitting [22] but also reduces the effect of overfitting [23]. After considering the specifics of the classified targets, a CNN architecture based on the VGG-net [24] is proposed for this inspecting method.

Purely in terms of a working principle, the purpose of training a deep learning system is to determine a set of weighting factors that is optimized to represent a detailed set of rules. Based on these rules, combined with an X-ray radiograph as the input data, the position of the key points can be estimated as the output values. This main task of training the deep learning model is preceded by a backpropagation process for establishing a suitable weighting factor in all layers. The weighting factor adjustment is calculated as the difference between the output ${A}_{j}$ from the deep learning model containing the calculated position value $j$ for the input image and the label ${Y}_{j}$ created based on the actual position of the required points in the input image. This difference is calculated with mean square error as the chosen loss function *L*, expressed in Eq. (1) with *n* outputs in the dataset.

1 $$L=\frac1n{\textstyle\sum_{i=1}^n}{(A_j-Y_j)}^2$$

As the difference between ${A}_{j}$ and ${Y}_{j}$ increases, the value of $L$ also increases. Optimization for the weighting factors ${w}_{q}^{p}$ between nodes *p* and *q* located in a neighboring layer is conducted based on the loss function difference in the direction from the output to input layer. After each training step, the weighting factor ${w}_{p}^{q}$ is updated by $\Delta {w}_{p}^{q}$, which can be derived from Eq. (2) with a learning rate *α*, determining the model learning speed [25]. Here, ${z}_{q}$ denotes a weighted input data summation by multiplying the weighting factor and ${o}_{q}$, which is the node output signal.

2 $$\Delta {w}_{p}^{q}=-\alpha \frac{\partial L}{\partial {o}_{q}}\frac{\partial {o}_{q}}{\partial {z}_{q}}\frac{\partial {z}_{q}}{\partial {w}_{p}^{q}}$$

During optimization of weighting factors, to calculate the difference between the current model performance and the labelled, exact values, the output calculated from the input and the weighting factors is necessary. This is called a feed forward process, in which the output ${\mathrm{Y}}^{\mathrm{n}}$ in layer n is calculated using Eq. (3) based on the input ${X}^{n}$ provided by the previous layer and the set of weighting factors ${w}_{i}^{n}$ in layer *n*, where ${b}_{i}^{n}$ is the bias, and $f$ is the activation function selected by the ReLU.

3 $$Y^n=f\left({\textstyle\sum_i}w_i^nX^n+b_i^n\right)$$

After this process is sequentially conducted to the final layer, the output is determined as the final predicted results ${A}_{j}$. Besides, the hyperparameters, which are initially set for training CNN model, include dropout values of 50%, Adadelta [26] optimizer for adjusting learning rate, training epoch number of 50, and chosen batch size of 32. Also, cross-validation is performed with 5th iterations with the ratio between training data and test data as 8:2, which has shown the similarity in term of model’s performance on test data.

## Results

### Validation Process

**Fig. 3:**
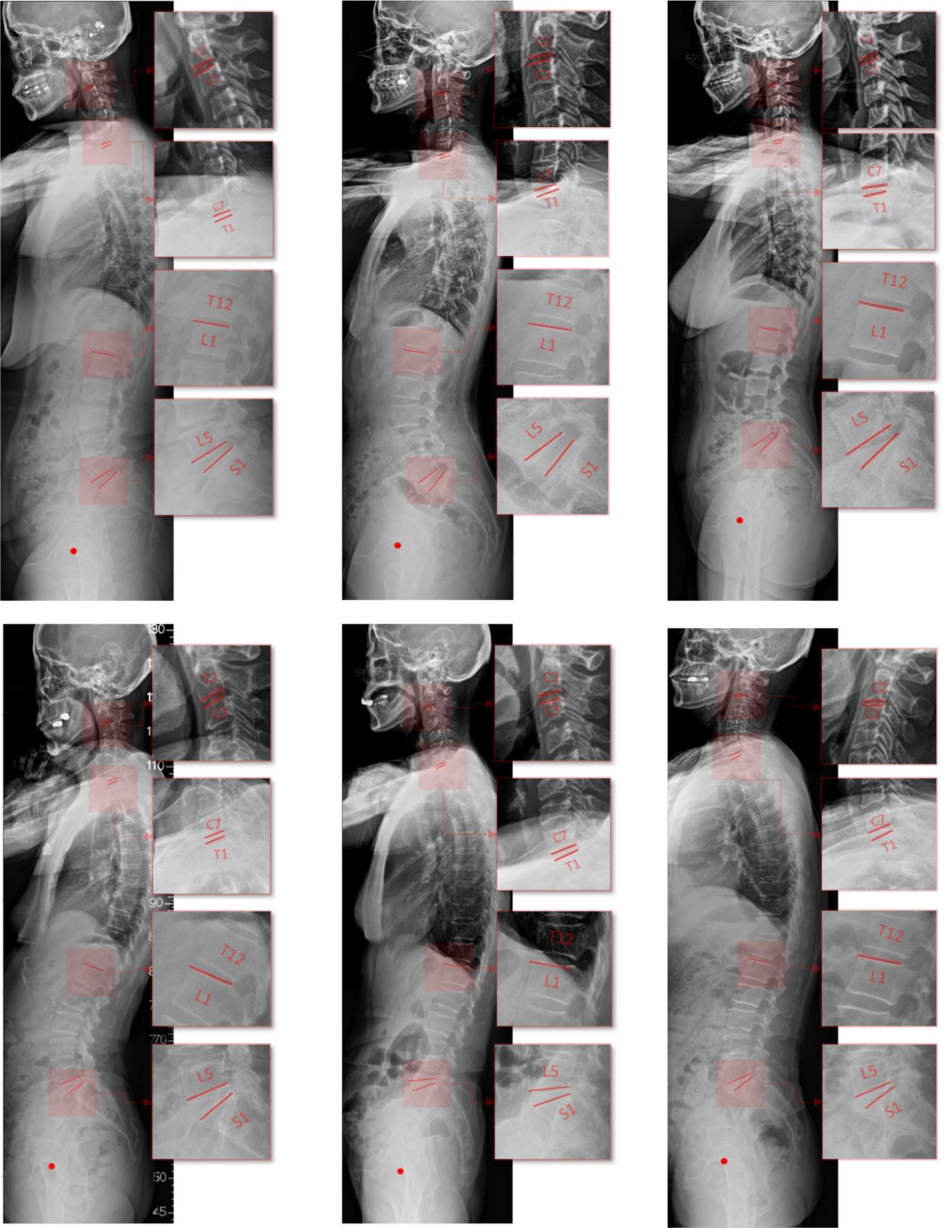
Example of detection results from test cases containing detected qualities of interest, including end plates of considered vertebrae and the center of femoral heads

To verify the performance of the proposed method, which was evaluated according to consistency with conventional manual measurements from experts, 30 spinal sagittal X-ray images that were not included in the training dataset were utilized. The considered alignment parameters of all test cases were manually measured by two experienced medical doctors and the mean values were determined as the standard references to ensure the reliability of the validation process. The automatic measurement of the proposed method, in which six examples of the 30 test cases are shown in Fig. 3, was compared to the standard references via comparison criteria of mean absolute differences and correlation coefficient.

### Comparison Results Between Proposed Method and Standard Reference

Based on the position of key points detected by the proposed method, 12 sagittal parameters were calculated. To validate the consistency between the performance of the developed method and the manual measurements of the experienced doctors, the correlation coefficient and coefficient of determination between two measurements were analyzed, as shown in Table 4. The method achieved the highest correlation in case of L1 incidence. However, the method appeared to have difficulties in measuring T1-related parameters, particularly the correlation coefficients of 0.584 for T1S and 0.751 for C27L. This difficulty also was observed in the mean absolute error (MAE) and standard deviation of absolute error (STD of AE), of which the lowest value was 1.156° for PT, and the largest error was from C27L.

**Table 4:** Correlation and deviation of spinal alignment parameters evaluated by CNN models and measured by experienced doctors

	PI	PT	SS	L1I	T1I	C2I	LL	TK	C27L	L1S	T1S	C2S
Corr. coefficient	0.968	0.984	0.893	0.983	0.823	0.935	0.958	0.836	0.751	0.957	0.584	0.860
*R*^2^	0.937	0.965	0.798	0.966	0.677	0.875	0.916	0.699	0.564	0.916	0.342	0.741
MAE (°)	2.205	1.156	3.171	2.252	5.842	3.355	3.895	5.737	6.318	2.128	6.241	3.708
STD of AE (°)	2.048	0.824	3.097	1.626	4.895	3.187	2.848	5.419	6.695	1.500	5.568	2.663

**Fig. 4:**
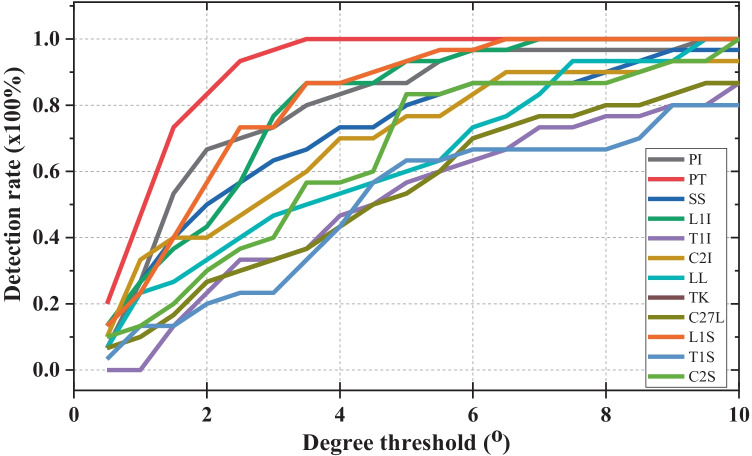
Detection rate for 12 parameters by degree of error threshold

To more clearly evaluate the performance of the proposed method with respect to each of the considered parameters, the detection rates of parameters, which is the ratio of the test case having the MAE of measured results smaller than the threshold, is enumerated, as shown in Fig. 4. The proposed method shows efficiency in measuring PT, L1I, L1S, and PI, with the detection rate for these angles reaching 0.8 with an error threshold of 3.5°, especially low at 2° in the case of PT. In addition, the visualized results of the T1-related parameters are incommensurate to the remaining results when they achieved a 0.8 detection rate at an error threshold of 8° in the C27L case and 9° for T1I and T1S.

**Fig. 5:**
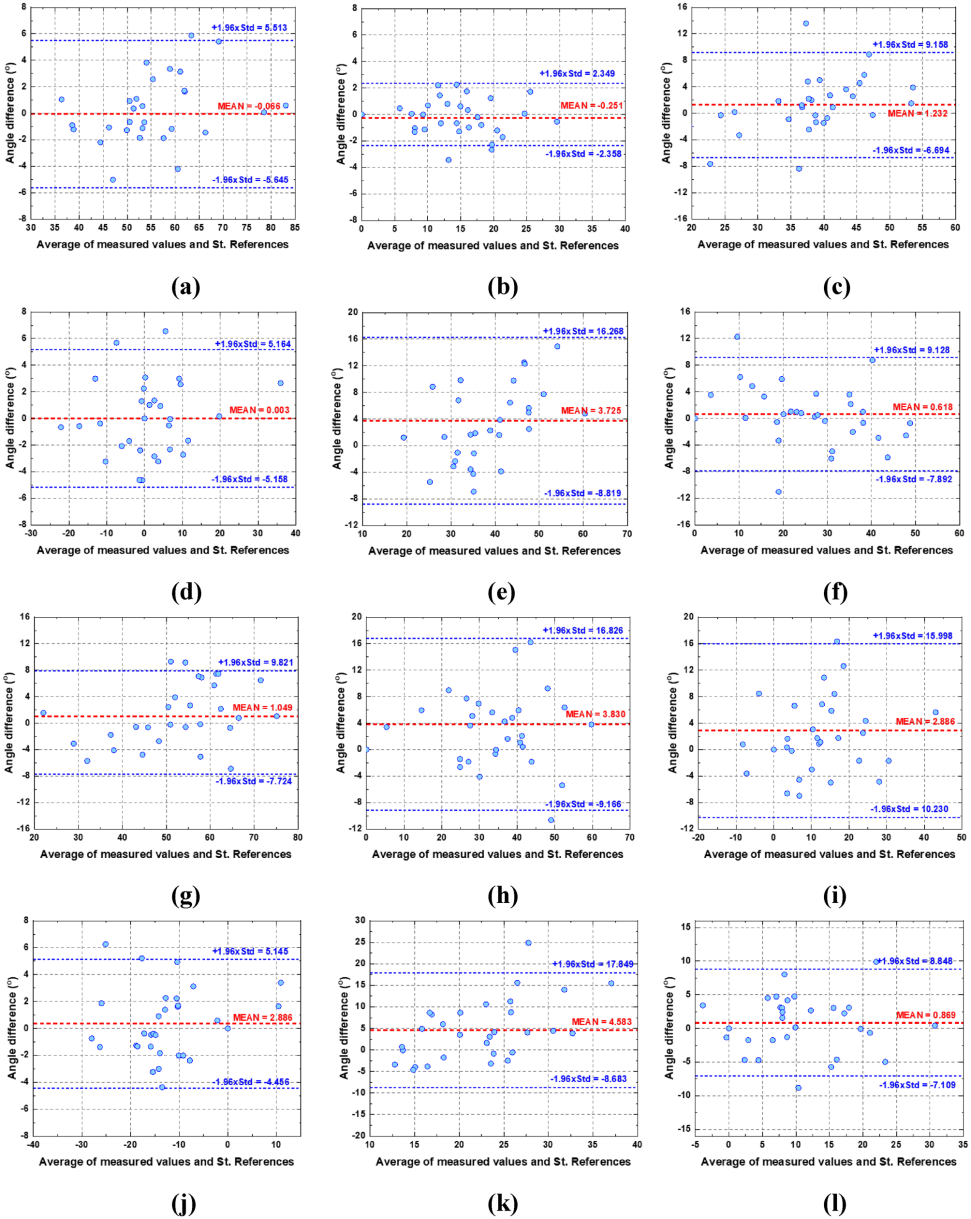
Bland–Altman plots comparing the values of radiographic parameters, including (**a)** PI, (**b)** PT, (**c)** SS, (**d)** L1I, (**e)** T1I, (**f)** C2I, (**g)** LL, (**h)** TK, (**i)** C27L, (**j)** L1S, (**k)** T1S, and (**l)** C2S

The test results of the method were visualized via Bland–Altman (B-A) plots for all angles of the entire sagittal parameters, as shown in Fig. 5, which includes the horizontal lines of mean difference (red solid line) and mean difference ± 1.96 standard deviation (SD) (blue dashed lines). The mean time required to perform the entire measurement procedure was less than 1 s.

To clarify the interobserver analysis from two experts and compare the variability between manual measurement and the proposed method, the correlation coefficient between values from two experts, between the automated measurement and each manual measurement, and also average of them, were calculated. Besides, the mean absolute error (MAE) and standard deviation (STD) of the absolute error were also considered. This comparison totally considered 360 angles consisting of 12 considered parameters in 30 validation cases as shown in Table 5.

**Table 5:** Comparison between measurement performed for validation

No	Measurement 1	Measurement 2	Corr. coefficient	MAE (°)	STD (°)
1	1st expert	2nd expert	0.9589	3.175	3.603
2	Proposed method	1st expert	0.9523	3.926	3.784
3	Proposed method	2nd expert	0.9527	3.988	3.789
4	Proposed method	Aver. of 1^st^ and 2nd exp	0.9558	3.832	3.369

About the metric of MAE and STD, it can be noticed that the MAE between two experts is smaller than between the proposed method and experts, including each expert separately and average of them. The reason of this point is that the proposed method’s results are similar to the 1st expert measurement in some cases and, in the other cases, to the 2nd expert measurement. Because the similarity of the proposed method and one of two manual measurements depends on cases, the error between the automated method and each expert, and also average of two experts is larger than error between experts. Therefore, if the error between the automated approach and manual measurement is considered as the minimum value between the error of automated approach and 1st expert, and the error of automated approach and 2nd expert, the MAE and STD between the automated approach and manual measurement, respectively, are 2.995 and 3.003°, which is equal to the MAE and STD between two experts.

## Discussion

This paper presents a method using a decentralized CNN to automatically determine spinal alignment by evaluating representative parameters from X-ray images. The proposed method is developed at the hierarchical levels of one model for locating ROIs of the cervical spine, lumbar spine, and femoral heads regions; a second model for positioning the landmarks considered; and a third model for determining the required key points. The advantages of this decentralization are due to the elimination of the effects of areas that are not necessary for detection by narrowing the considered areas at each successive model level based on the geometrical characteristics of the spine. Detection of key points that have geometrical characteristics, such as corners of vertebrae and center circles of femur heads, with small ROIs in the third model shows high accuracy. In addition, the ability to expand the dataset diversity by utilizing vertebral images not included in the image of the entire spine contributes to the quality of this approach.

From the perspective of the medical field, this artificial intelligence (AI)-based measurement method possesses highly accurate automated ROI and key point detection rates. Compared to the expert-dependent manual measurement method, this AI-based method has the potential for nearly infinite expansion in its scope of technological applications. The knowledge of spinal sagittal alignment has increased linearly through manual measurement of preoperative and postoperative sagittal parameters in patients requiring surgery. However, the AI-based measurement method will enable an exponential increase in the knowledge of the spinal alignment by rapidly analyzing the chronological and reciprocal change of the spinal alignment of degenerative spondylotic patients as well as the normal population. In addition, the accumulated knowledge will help determine the degree of optimum deformity correction of the spine using a deep mining algorithm to compare patients with good versus bad prognoses.

Segmentation of X-ray images or reconstruction of three-dimensional (3D) profiles utilizing the deep learning technique [9, 11] are alternative potential directions for use of X-ray images. However, for the measurement results to be applied for diagnosis and as design parameters of specialized treatment equipment [27–31], accuracy is a key factor that should be considered as a top priority. Training a highly accurate segmentation or 3D reconstruction task model requires a diverse set of data, a challenge in this field. In addition, poor visual quality of images, which depends on facilities and is difficult to synchronize, has been cited as a limitation. In contrast, focusing on identifying key points as the geometric characteristics of the proposed method has succeeded in limiting the impact of image quality. This is highlighted by comparison of mean errors of the proposed algorithm (2.93° in LLA) with performance of a method in [9] (3.6° in LLA) and a method in [11] (8.055° in LLA).

There are other research approaches that propose deep learning models designed based on location of key points. After comparing the accuracy of these studies, the proposed method provides a significant advantage. These results compare favorably to those of a state-of-the-art method [32] for automatic Cobb angle prediction using deep learning, which reported an MAE of 11.5°, 9.5°, 8.5°, and 2.7° in LL, PI, SS, and PT cases, respectively. That advantage is confirmed by comparing the performance of the method of [12], which had greater accuracy, with an MAE of 4.04° for Cobb angle estimation. The utilization of a decentralized CNN method [13, 14] provides a substantially lower deviation between automatic detection and direct measurement from a medical doctor. This deviation is 1.76° with respect to segmental motion angle and 3.17°, 3.53°, 2.64°, 1.45°, and 2.51° for LL, lumbar sacral joint angle, PT, PI, and SS, respectively.

**Fig. 6:**
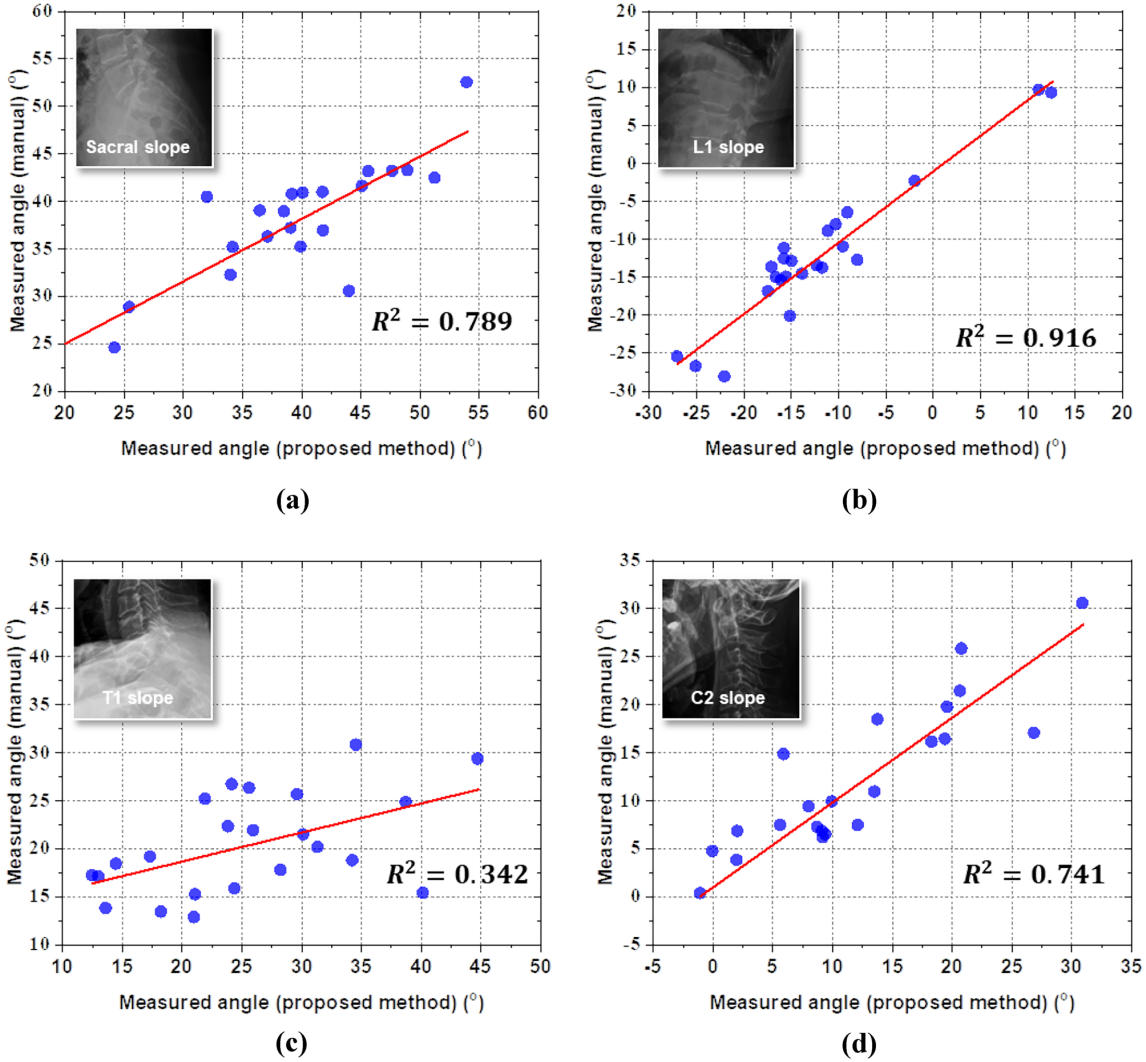
Scatterplots of correlation between standard references and the proposed methods with respect to (**a)** SS, (**b)** L1S, (**c)** T1S, and (**d)** C2S

However, the proposed method has two main limitations. First, utilization of a decentralized CNN requires separate datasets which are contributed for each order and consequently consume time not only for creating the dataset but also for comprehensively training the CNN models. Second, there is a significant obstacle related to the lack of clarity of conventional X-ray images in the ROIs of T1 vertebrae. To visualize deviation of the performance of the proposed method by parameter, the correlations between manual measurements and obtained results of SS, L1S, T1S, and C2S are shown in Fig. 6. All four angles are measured between the specific lines from the detected key points and the horizontal axis, which ensures the equivalent dependence of accuracy on the measured results. Based on the graphs shown, there seems to be a significant gap of method performance between angles, particularly the 0.584 correlation coefficient of T1S and the remaining parameters.

These issues might be caused by a characteristic of the shoulder girdle of human, which usually overlaps on the spinal regions in sagittal X-rays. Therefore, visualization of the last vertebrae in the cervical region and the upper half of the thoracic region generally contains significant noise compared to other regions. The noise not only limits the performance of the proposed method but also causes difficulties for manual implementation.

About the clinical significance of errors, considering the high correlation and small error in most of parameter cases, except the T1 vertebrae-related parameters, it is considered acceptable, especially with the purpose of this method which is to provide doctors the global deformity of spine for diagnosing and establishing the treatment. Besides, the most significant advantage of this approach comparing to manual measurement is reducing amount of time consumed by detecting features of vertebras by hand, which decreases from around 10 min for one case to less than 1 s.

Consequently, the second limitation opens a future research direction on enhancing the visual quality of X-ray images. To deal with this limitation, the future research direction utilizing image-to-image deep learning, which receive the sagittal X-rays of T1 region as the input and provide a de-blurred image with high clarity of vertebrae features for experts and automated method to measure, is now being considered.

## Conclusion

Based on the time and effort required of an experienced doctor to measure alignment parameters of the spine, development of an automated measurement software that can utilize deep learning and leverage the rapid advancement of big data technologies could free labor from repetitive work and increase productivity because of its ability to process a large amount of data. A fully automatic method was proposed for measuring 12 spinal alignment parameters, PI, PT, SS, L1I, T1I, C2I, LL, TK, C27L, L1S, T1S, and C2S using a designed decentralized CNN algorithm. To validate the performance of the proposed method, the measured results of 30 test cases were compared to standard references created by human experts. The quality of the proposed method, which was evaluated in terms of the correlation coefficient and MAE, was recorded with good results for 10 of 12 parameters, while the gap of accuracy of the two remaining parameters was determined to be an effect of inevitable local noise in the images. In addition, the calculation time was less than 1 s for all cases. Because of difficulties in measuring parameters under in noisy conditions, future research will focus on the reconstruction of images with enhanced visual conditions using image-to-image deep learning techniques.

## Data Availability

Not applicable.

## References

[1] Le Huec J, C., Saddiki, R., Franke, J.,  (2011). Equilibrium of the human body and the gravity line: the basics. European Spine Journal.

[2] Been E, Kalichman L (2014). Lumbar lordosis. The spine journal.

[3] Briggs AM, van Dieen JH, Wrigley TV (2007). Thoracic Kyphosis Affects Spinal Loads and Trunk Muscle Force. Physical Therapy.

[4] Been E, Shefi S, Soudack M (2017). Cervical lordosis: the effect of age and gender. The Spine Journal.

[5] Kim HJ, Yang JH, Chang D (2020). Adult Spinal Deformity: Current Concepts and Decision-Making Strategies for Management. Asian Spine Journal.

[6] Borden AGB, Rechtman AM, Gershon-Cohen J (1960). The normal cervical lordosis. Radiology.

[7] Fon GT, Pitt Cole MJ, Thies Jr, A: Thoracic Kyphosis: Range in normal subjects. Am J Roentgenol 134:979-983,1980 10.2214/ajr.134.5.979 6768276

[8] Russell P, Pearcy MJ, Unsworth A (1993). Measurement of The Range and Coupled Movements Observed in The Lumbar Spine. Rheumatology.

[9] Aubert B, Vazquez C, Cresson T, Parent S, de Guise JA (2019). Toward Automated 3D Spine Reconstruction from Biplanar Radiographs Using CNN for Statistical Spine Model Fitting. IEEE Transactions On Medical Imaging.

[10] Weng C, H., Wang, C. L., Huang, C. L., Yeh, Y. C., Fu, C. J., Yeh, C. Y., Tsai, T. T.  (2019). Artificial Intelligence for Automatic Measurement of Sagittal Vertical Axis Using ResUNet Framework. Journal of Clinical Medicine.

[11] Cho BH, Kaji D, Cheung ZB, Ye IB, Tang R, Ahn A, Carrillo O, Schwartz JT, Valliani AA, Oermann EK, Arvind V, Ranti D, Sun L, Kim JS, Cho SK: Automated Measurement of Lumbar Lordosis on Radiographs Using Machine Learning and Computer Vision. Glob Spine J 1–8,2019 10.1177/2192568219868190 PMC7359685 32677567

[12] Wu H, Bailey C, Rasoulinejad P, Li S (2018). Automated comprehensive Adolescent Idiopathic Scoliosis assessment using MVC-Net. Medical Image Analysis.

[13] Chae DS, Nguyen TP, Park SJ, Kang KY, Won C, Yoon J (2020). Decentralized convolutional neural network for evaluating spinal deformity with spinopelvic parameters. Computer Methods and Programs in Biomedicine.

[14] Nguyen TP, Chae DS, Park SJ, Kang KY, Yoon J (2021). Deep learning system for Meyerding classification and segmental motion measurement in diagnosis of lumbar spondylolisthesis. Biomedical Signal Processing and Control.

[15] Choi S, H., Hwang, C. J., Cho, J. H.,  (2020). The influence of spinopelvic morphologies on sagittal spinal alignment: an analysis of incidence angle of inflection points. European Spine Journal.

[16] Fukushima K (1980). Neocognitron: A Self-Organizing Neural Network Model for a Mechanism of Pattern Recognition Unaffected by Shift in Position. Biological Cybernetics.

[17] Wang P, Chen P, Yuan Y, Liu D, Huang Z, Hou X, Cottrell G (2018). Understanding Convolution for Semantic Segmentation. IEEE Winter Conference on Applications of Computer Vision (WACV).

[18] Nagi J, Ducatelle F, Caro GAD, Ciresan D, Meier U, Giusti A, Nagi F, Schmidhuber J, Gambardella LM: Max-Pooling Convolutional Neural Networks for Vision-based Hand Gesture Recognition. 2011 IEEE International Conference on Signal and Image Processing Applications (ICSIPA 2011), 342–347,2011

[19] Dahl GE, Sainath TN, Hinton GE: Improving deep neural networks for LVCSR using rectified linear units and dropout. 2013 IEEE International Conference on Acoustics, Speech and Signal Processing 8609–8613,2013

[20] Srivastva N, Hinton GE, Krizhevsky A, Sutskever I, Salakhutdinov R (2014). Dropout: A Simple Way to Prevent Neural Networks from Overfitting. Journal of Machine Learning Research.

[21] Hinton GE, Krizhevsky A, Sutskever I, Srivastva N: System and method for addressing overfitting in a neural network. US patent 9,406,017. Washington, DC: U.S. Patent and Trademark Office. 2016

[22] Narayang S, Tagliarini G: An analysis of underfitting in MLP networks. Proceedings. 2005 IEEE Int Joint Conf Neur Netw 984–988,2005

[23] Hawkins D, M.  (2004). The Problem of Overfitting. Journal of Chemical Information and Modeling.

[24] Simonyan K, Zisserman A: Very deep convolutional networks for large-scale image recognition in ICLR. 2015

[25] Madhavan S, Tripathy RK, Pachori RB (2020). Time-Frequency Domain Deep Convolutional Neural Network for the Classification of Focal and Non-Focal EEG Signals. IEEE Sensors Journal.

[26] Zeiler MD: Adadelta: An adaptive learning rate method. arXiv preprint. 2012

[27] Polly DW, Kikelly FX, McHale KA, Asplund LM, Mulligan M, Chang AS: Measurement of lumbar lordosis. Evaluation of intraobserver, interobserver, and technique variability. Spine 21(13):1530–1536,1996 10.1097/00007632-199607010-00008 8817780

[28] Chernukha KV, Daffner RH, Reigel DH: Lumbar lordosis measurement. A new method versus Cobb technique. Spine 23(1):74–80,1998 10.1097/00007632-199801010-00016 9460156

[29] Cobb J (1948). Outline for the study of scoliosis. Instructional course lectures.

[30] Guo H, Zhuang X, Rabczuk T (2019). A Deep Collocation Method for the Bending Analysis of Kirchhoff. Computers, Materials & Continua.

[31] Samaniego E, Anitescu C, Goswami S, Nguyen-Thanh VM, Guo H, Hamdia K, Zhuang X, Rabczuk T: Comput Methods Appl Mech Eng 362:112790,2020

[32] Galbusera F, Niemeyer F, Wilke HJ, Basani T, Casaroli G, Anania C, Costa F, Bruno MB, Sconfienza LM (2019). Fully automated radiological analysis of spinal disorders and deformities: a deep learning approach. European Spine Journal.

